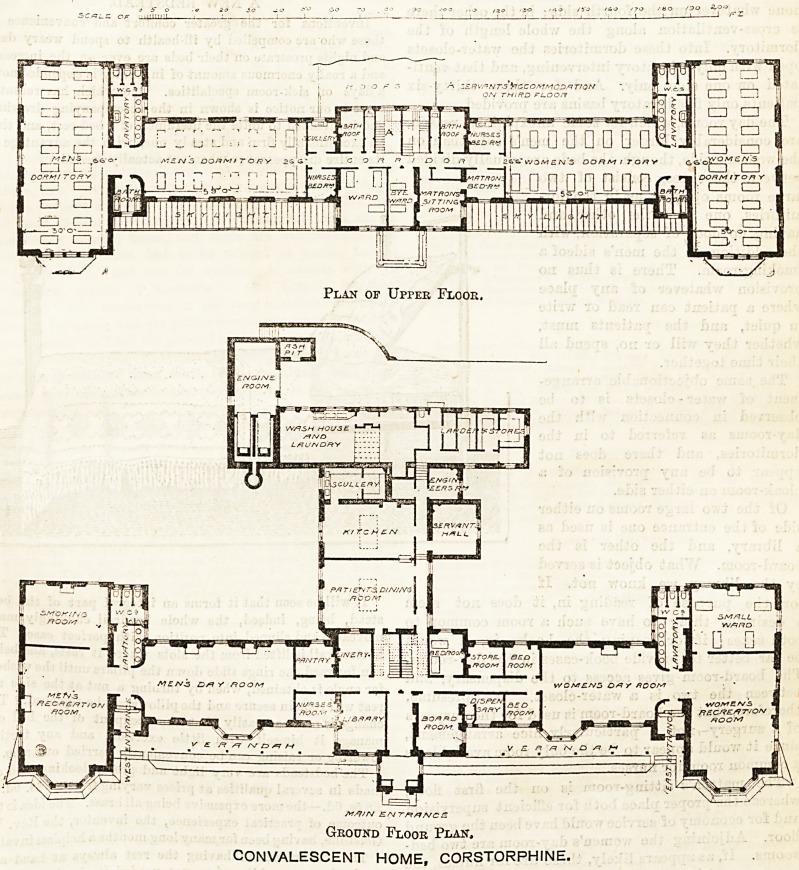# The Convalescent Home of the Royal Infirmary of Edinburgh, Corstorphine

**Published:** 1894-08-04

**Authors:** 


### The Institutional Workshop.

### HOSPITAL CONSTRUCTION.

### THE CONVALESCENT HOME OF THE ROYAL INFIRMARY OF EDINBURGH, CORSTORPHINE.

This institution was opened for patients in the year 1867 with accommodation for sixty patients, and some wings at either end, and a one storey wing at the back.

The central block is mainly occupied with administrative offices and quarters for the staff; the right wing is for women patients, the left wing for men, and the wing at the back contains the common dining-room and the kitchen offices and laundry.

Plan of Upper Floor.

Ground Floor Plan.

CONVALESCENT HOME, CORSTORPHINE.

twenty-five years later, with the proceeds of a legacy from Mr. James Nasmyth, the well-known engineer, the managers were able to add two wings to the original structure and thus increase the total number of beds to one hundred. As will be seen from the plans which we publish to-day, the building consists of a long two storey front building with projecting

The principles upon which this building has been designed appear to us to be in direct opposition to almost every rule which should govern the planning of a convalescent home.

To begin with the dormitories. There is no advantage to be gained in massing convalescent patients into large wards; on the contrary, it is distinctly desirable both for the comfort of the patients and for the discipline of the place that they should be grouped together in moderate sized rooms with plenty of air space; but it is not at all necessary or desirable that dormitories occupied as a rule by night only should be provided with the ample through ventilation necessary for sick wards which are occupied continuously night and day. We find here on each side two large dormitories, opening one into the other, and containing together no less than thirty-six patients. In one case there is ventilation on three sides, but none whatever on the fourth side; in the other there is cross-ventilation along the whole length of the dormitory. Into these dormitories the water-closets open with only a lavatory intervening, and that ventilated on one side only. And for these thirty-six patients only five lavatory basins are provided.

The day rooms, which for some unexplained reason, are considerably larger on the men's side than on the women's side, though the beds are equally divided between the sexes, consist of two large rooms opening, as in the dormitories, one into the other and having no door of separation, with the addition on the men's sideof a smoking-room. There is thus no provision whatever of any place where a patient can read or write in quiet, and the patients must, whether they will or no, spend all their time together.

The same objectionable arrangement of water-closets is to be observed in connection with the day-rooms as referred to in the dormitories, and there does not appear to be any provision of a cloak-room on either side.

Of the two large rooms on either side of the entrance one is used as a library, and the other is the board-room. What object is served by the library, we know not. If for the purpose of reading in, it does not seem a desirable thing to have such a room common to both sexes; if for keeping the books in, it would be far better to provide book-cases in the day-rooms. The board-room gives access to the dispensary, and between the two is a water-closet. We presume, therefore, that the board-room is used for the purposes of a surgery—not a particularly nice arrangement, since it would appear to be the only room available as a common room for nurses.

The matron's sitting-room is on the first floor, whereas the proper place both for efficient supervision and for economy of service would have been the ground floor. Adjoining the women's day-room are two bedrooms. If, as appears likely, these are for nurses, the arrangement is a most undesirable one.

The patients' dining-room appears as much too small as the day-rooms are excessive in size, and the arrangement for serving meals direct from the kitchen without the intervention of a serving-room is not a good one, and tends to confusion in the kitchen.

Finally, there does not appear to be any provision for isolating cases of infectious disease if occasion should arise.

We regret to have been compelled to criticize so severely a building which must, in spite of its many defects, be a most valuable adjunct to the famous hospital to which it belongs, but it has rarely been our lot to describe plans which exemplify so emphaticallyt he exact converse of what they should be.

## Figures and Tables

**Figure f1:**